# Unveiling the Hidden Therapeutic Potential of Carnosine, a Molecule with a Multimodal Mechanism of Action: A Position Paper

**DOI:** 10.3390/molecules27103303

**Published:** 2022-05-20

**Authors:** Giuseppe Caruso

**Affiliations:** 1 Department of Drug and Health Sciences, University of Catania, 95125 Catania, Italy; giuseppe.caruso2@unict.it; Tel.: +39-095-7385036; 2 Unit of Neuropharmacology and Translational Neurosciences, Oasi Research Institute—IRCCS, 94018 Troina, Italy

**Keywords:** carnosine, therapeutic potential, preclinical studies, doses and administration routes, treatment duration, animal models

## Abstract

Carnosine (β-alanyl-L-histidine) is a naturally occurring endogenous dipeptide and an over-the-counter food supplement with a well-demonstrated multimodal mechanism of action that includes the detoxification of reactive oxygen and nitrogen species, the down-regulation of the production of pro-inflammatory mediators, the inhibition of aberrant protein formation, and the modulation of cells in the peripheral (macrophages) and brain (microglia) immune systems. Since its discovery more than 100 years ago, a plethora of in vivo preclinical studies have been carried out; however, there is still substantial heterogeneity regarding the route of administration, the dosage, the duration of the treatment, and the animal model selected, underlining the urgent need for “coordinated/aligned” preclinical studies laying the foundations for well-defined future clinical trials. The main aim of the present position paper is to critically and concisely consider these key points and open a discussion on the possible “alignment” for future studies, with the goal of validating the full therapeutic potential of this intriguing molecule.

## 1. Carnosine: History and Biological Activities

Carnosine (β-alanyl-L-histidine) was discovered by Gulewitsch and Amiradžibi (Laboratorium der Universität Charkow, Charkow, Ukraine) more than 100 years ago during a study in which they were analyzing a meat extract [1]. They isolated and characterized several unidentified (at that time) nitrogen-containing compounds, one of which was carnosine. Since this molecule was isolated from minced meat, it was given the name “carnosine”, from the Latin *caro*, *carnis* (meat).

Carnosine is a naturally occurring endogenous dipeptide synthesized by the enzyme carnosine synthase 1 (CARNS1) from its constituent amino acids, β-alanine (synthesized in the liver) and L-histidine (external source) [2,3]. The levels of this dipeptide are very high (millimolar order of magnitude) in cardiac and skeletal muscles (∼99% of the carnosine in the body) [4,5] as well as in the brain [6]. Carnosine levels in human tissues and biological fluids are regulated by the activity of two carnosinases, enzymes that are able to decompose it into β-alanine and L-histidine: serum-circulating carnosine dipeptidase 1 (CNDP1) [7] and cytosolic carnosine dipeptidase 2 (CNDP2) [8], which belong to the M20 metalloprotease family [9]. A variety of other histidine-containing dipeptides such as anserine and balenine, methylated analogues of carnosine, can be found in the tissues of several animal species [10].

The biological activities and potential applications of carnosine are widespread and noteworthy. Although the muscles and brain contain the highest levels of carnosine, this dipeptide also performs biological activities in other areas of the body. Over the last 70 years, more than a thousand papers have been published describing the structure of carnosine and its biological activities in different systems; in particular, numerous studies have investigated the physiological role played by carnosine in muscles, probably due to its predominant localization in these areas, and the benefits of its exogenous supplementation for athletic performance enhancement. In this context, the supplementation of carnosine or β-alanine has been shown to improve the strength of muscle contraction and the mechanical work produced (“Severin’s phenomenon”) [11]; reduce the accumulation of lactate in active muscles, thus preventing intramuscular acidification [12]; increase the contraction and relaxation rates of muscles [13]; activate contractile proteins and stabilize muscles’ energy metabolism [14]; and improve physical performance and executive function following endurance exercise [15,16,17,18].

As previously mentioned, the biological effects and potential benefits of carnosine are not limited to muscle tissue. In fact, this dipeptide has shown the ability to act as a neurotransmitter [19], an enhancer of cell energy metabolism [20,21] and the immune system [22], a modulator of the metabolism of nitric oxide (NO) and related species [23,24,25], an anti-glycation and anti-aging agent [26,27], and a chelator of heavy metals [28,29]. Additionally, carnosine can modulate the glutamatergic system through the up-regulation of glutamate transporter 1 and the reduction of glutamate levels in the central nervous system (CNS) [30].

Taking into consideration all the above-mentioned activities of carnosine, it is clear why numerous research groups are working on carnosine and believe in its high therapeutic potential. In this regard, I would like to give credit to the enormous efforts made by Alan Hipkiss during the last three decades in describing the potential of carnosine and, in particular, how some of its activities could be useful for the treatment of cancer [10], Parkinson’s disease (PD) [31], depression, diabetes, dementia [32], Alzheimer’s disease (AD) [33], and COVID-19 [34]. Several other authors have contributed significantly to the advancement of our knowledge on the role of carnosine, including Giancarlo Aldini [4,35,36,37], Wim Derave [4,38,39], Craig Sale [40,41,42], Barbora De Courten [37,38,43], Guilherme Artioli [40,41,42], and Alexander Boldyrev [4,44].

## 2. The Multimodal Mechanism of Action of Carnosine: Contribution from In Vitro Studies

Sometimes it seems that there is a clear separation between those who believe in in vitro studies and those who believe only in in vivo research and consider the latter to be the most important step for the transition to studies on human beings; in vitro studies have developed a reputation for being “less translatable” to humans. The truth is that both are interrelated and indispensable to fully understanding the therapeutic potential of a molecule of interest. In vitro studies have several advantages over in vivo research; for example, they allow the tight control of the chemical and physical environment; reduce the research costs; provide the opportunity to obtain a higher throughput; and minimize the use of animals, which is nowadays strongly limited by the growing ethical concerns. Additionally, as has been demonstrated in carnosine research but is applicable to scientific research as a whole, in vitro experiments allow the in-depth study of the different mechanisms of action related to a specific phenomenon, which is often very difficult or only partially possible in vivo.

There is continually increasing evidence that inflammation [45,46,47], oxidative stress [48,49,50], and aberrant aggregation and accumulation of proteins [51,52] significantly contribute to numerous systemic and neurodegenerative disorders such as diabetes mellitus type 2 (T2DM) [53,54], PD [55,56], and AD [57,58,59]. In this context, the well-known and frequently cited anti-oxidant, anti-inflammatory, and anti-aggregant activities of carnosine, which underline its multimodal mechanism of action, have been considered.

A plethora of in vitro studies have shown the various protective activities of carnosine in multiple heterogeneous cell types, such as macrophages/microglia [21,60,61,62], myocytes [63], skeletal muscle myoblasts [64], podocytes [65], endothelial cells [66], pancreatic β-cells [67], chondrocytes [68], fibroblasts [69], hepatic cells [70], lymphocytes [71], erythrocytes [72], astrocytes [30,73], neuron-like cells [74,75], and stem cells [76]. The “coverage” of the very wide range of cell types demonstrated in vitro, representative of different body districts, strengthens the idea that carnosine has the potential to exert therapeutic effects in a broad spectrum of pathological conditions.

Despite the considerable number of in vitro experiments that have been performed and the diversity of the cell types considered, similar concentrations of carnosine (millimolar order of magnitude) and durations of treatment (often within 24 h) have been used in most studies, allowing the results to be reproduced and compared and establishing a common basis for in vivo experiments.

I will now discuss the therapeutic potential of carnosine as demonstrated in numerous clinical trials, in which greater heterogeneity, specifically in terms of methodology, can be observed compared to the in vitro studies.

## 3. Clinical Trials of Carnosine: What Is the Basis for the Heterogeneity?

According to www.clinicaltrials.gov (accessed on 15 April 2022), a service provided by the U.S. National Institute of Health that shares information on current clinical trials, there are currently 32 studies at different stages (not yet recruiting, recruiting, completed, unknown, etc.) using carnosine, its analog zinc L-carnosine (polaprezinc [77]), β-alanine, or carnosine-rich foods for the treatment of various diseases (peripheral arterial disease, bipolar I disorder, schizophrenia, AD, multiple sclerosis, etc.).

In addition to the above, numerous clinical trials have been conducted to explore the therapeutic effects of carnosine in a wide range of diseases/pathological conditions (including age-related conditions). For example, Gulf War illness (carnosine 1500 mg/day; 12 weeks (dose-escalation study)) [78]; mild cognitive impairment (MCI) (anserine:carnosine 750 mg:250 mg/day; 12 weeks) [79]; health status of elderly people treated with a pill-based nutraceutical (NT-020) containing carnosine (unspecified dosage; 2 months) [80], a formulation (formula F) containing carnosine (100 mg; 6 months) [81], or carnosine:anserine (250–350:650–750 mg/day; 13 weeks) [82,83,84]; depression (carnosine 400 mg twice daily; 6 weeks) [85]; T2DM (carnosine 1 g/day; 12 weeks) [86]; binge eating disorder and bulimia nervosa (polaprezinc 150 mg/day: 34 mg zinc and 116 mg L-carnosine; 16 weeks); autism spectrum disorder (carnosine 10–15 mg/kg/day plus standard care treatment; 2 months) [87]; and attention-deficit/hyperactivity disorder (carnosine 800 mg/day; 8 weeks [88]. As can be seen, carnosine has been co-administered with anserine in multiple studies. This natural derivative of carnosine, with equivalent physiological functions [4], is usually adopted because it is not cleaved by human carnosinase, which is abundant in human serum and is known to strongly reduce carnosine bioavailability [89].

One of the reasons why so many studies have been carried out on carnosine may be because both preclinical [90] and clinical [91,92] studies have demonstrated that this dipeptide is essentially non-toxic and well-tolerated, without any known drug interactions or dangerous side effects.

A detailed analysis of the clinical trials reveals substantial heterogeneity among the studies carried out on carnosine, alone or in combination with other molecules. Inevitably, studies will differ in terms of both clinical heterogeneity, due to the variability of participants, interventions, and outcomes, and methodological heterogeneity, due to the differences in the study designs and risk of bias. As a concrete example, my colleagues and I recently published a systematic review with meta-analysis on the therapeutic potential of carnosine/anserine supplementation against cognitive decline [93]. Despite the reasonably high number of studies that resulted from the systematic search (516), only 36 were still considered after an initial (i.e., title and abstract) evaluation. Unfortunately, 31 of these 36 studies did not meet the pre-specified inclusion criteria; we were forced to exclude most of the articles because they: (a) reported the acute effects of carnosine supplementation; (b) included results from children; (c) reported other outcomes (e.g., quality of life or cognitive performance); (d) did not explore the outcomes of interest; or (e) were partially conducted on the same patients. In the end, we included only five studies [79,81,83,94,95] in the systematic review, and only three provided sufficient statistical data to be included in the quantitative analysis.

At this point, we should carefully and critically consider what lies “between” in vitro studies and clinical trials, that is, in vivo research, which is the truly pivotal stage.

## 4. In Vivo Preclinical Studies: Administration Route, Dosage, Treatment Duration, and Selected Animal Model. Are We All Converging in the Same Direction?

Despite the limits imposed by ethical concerns, the coupling of in vivo and in vitro experiments is of utmost importance for fully understanding the therapeutic potential of a candidate molecule. The use of animals provides an opportunity to addresses many of the shortcomings of in vitro studies, particularly by allowing a more accurate evaluation of the safety, toxicity, and efficacy in a complex model. Additionally, advances in the “modulation of animal genotypes” have helped researchers to replicate human diseases with very high accuracy.

**Figure 1:**
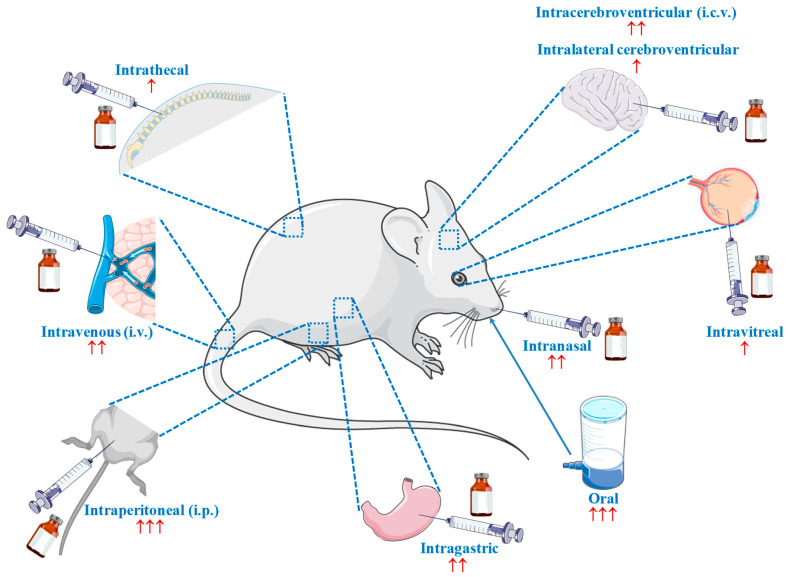
Different routes employed for the administration of carnosine in vivo. **↑** = rarely used (1 to 2 times); **↑↑** = infrequently used (3 to 6 times); **↑↑↑** = often used (>50 times). Part of the figure was generated using Servier Medical Art, available at smart.servier.com (accessed on 20 April 2022).

Since the publication of Tomonaga et al. in 2004 [96], which investigated the effect of the central administration of carnosine and its constituents on the behavior of chicks, more than 140 in vivo research studies have been published describing the therapeutic potential of carnosine for numerous diseases, such as stroke [97], diabetes [98], depression [99], liver injury [100], hypoxia–ischemia [101], AD [102], dyslipidaemia [103], atherosclerosis [104], myocardial infarction [105], PD [106], septic shock [107], autism spectrum disorder [108], and autoimmune encephalomyelitis [109]. This incomplete list of disorders is enough to clearly illustrate the substantial attention that this molecule has garnered. At the same time, the great variability of the route of carnosine administration stands out as a crucial point that deserves to be discussed. Researchers have tested many different administration routes (and related formulations), including intranasal, oral, intracerebroventricular (i.c.v.), intraperitoneal (i.p.), intravenous (i.v.), intralateral cerebroventricular, intravitreal, intragastric, and intrathecal (Figure 1), with oral and i.p. being the most widely employed.

One might assume that this heterogeneity arose due to the “physiological variability” of the different animal models; however, substantial discrepancies can be found not only within the same animal model, but also within the same strain, as in the case of C57BL/6 mice, for which i.p. [110], oral [111], and i.v. [112] administration routes have been used. Furthermore, this situation highlights a major unmet need in the in vivo research on carnosine—the lack of studies on its bioavailability after a specific administration process. Often, we are only able to observe the endpoint effects, which cannot be correlated to a specific carnosine concentration in a specific area of the body. The implementation of pharmacokinetic studies showing how a specific administration route influences the quantity of the molecule that reaches specific organs and tissues will help us understand more comprehensively the therapeutic potential of carnosine and evaluate more accurately the results already obtained by research groups. Supposing that we manage to administer a certain amount of carnosine into the brain, the next question is “how much carnosine will be able to overcome a selective barrier such as the blood–brain barrier (BBB)?”.

Two additional critical factors, characterized by substantial variability and related “comparison/translational” issues, are dosage and treatment duration. It can be expected that some of the differences observed according to dosage are caused by the administration route selected; for example, “mg/kg of body weight” is often used for i.p. [100,113,114], while “g/L” is frequently used for oral administration through drinking water [115,116,117]. Nevertheless, as observed in the case of animal strains, there is also a source of “internal” variability that complicates the comparison of results between different studies. In studies involving oral (drinking water) administration, one of the two “often used” (Figure 1) administration routes, the dosage amounts vary widely, ranging from 0.5 g/L [98,116] to 30 g/L (60 times higher!) [109,118]. Furthermore, in the context of oral administration, at least five different units of measurement can be found within the research studies: mg or g/Kg [119,120,121], mg or g/L [98,115,116], mmol/L or Kg [99,122,123,124], mM [102,125,126], or % [127,128,129]. This, along with the wide range of treatment durations (from 1 [120] to 50 weeks [130]), reinforces the need for the alignment of future studies, which will likely lead to even more surprising results with regard to the therapeutic potential of carnosine.

**Figure 2:**
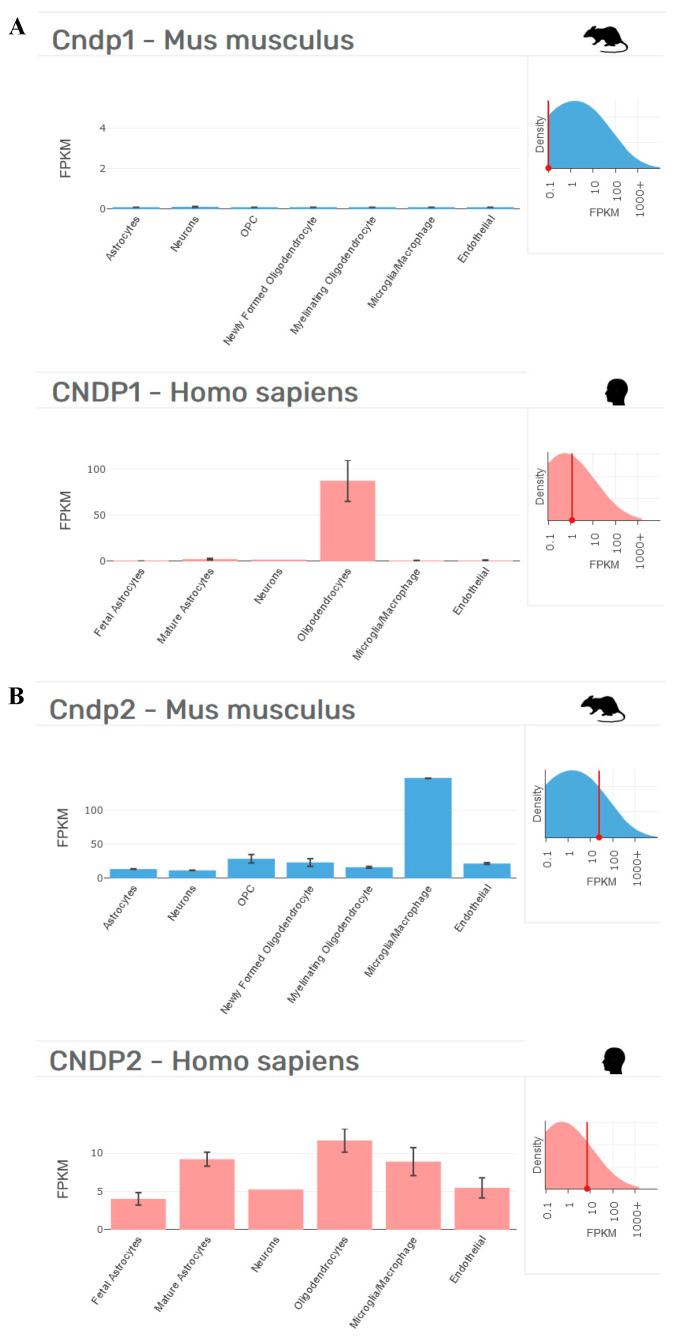
Cellular distribution in the brains of *Mus musculus* and *Homo sapiens* of (**A**) CNDP1 and (**B**) CNDP2, according to transcriptome studies [131,132]. Fragments per kilobase of transcript per million mapped reads (FPKM) data collected from http://www.brainrnaseq.org on 17 April 2022.

When performing in vivo studies, an additional drawback could be represented by the animal model selected. In this context, it is worth recalling that the administration of carnosine in humans only leads to a small increase in circulating carnosine. When we consider both the bioavailability and high therapeutic potential of carnosine in rodents (mice and rats) and humans, it is important to consider that rodents lack the signal peptide in the CNDP1 gene (CTG)_5_ and consequently do not have circulating CNDP1 enzymes [4] (see Figure 2 for an example).

These interspecies differences could explain why the levels of carnosine increase in rodents (>120 studies carried out since 2005) after oral carnosine supplementation [125], a phenomenon that does not occur in humans, who only experience a small increase in circulating carnosine [133]. The results obtained in rodents could represent an overestimation of the therapeutic potential of carnosine that will not be confirmed by a translational approach, moving from mice/rats to humans.

Consequently, different research groups are currently working on the development of new approaches and new formulations of carnosine that are able to improve its bioavailability and/or reach a specific target (drug delivery systems). One potential approach to increasing carnosine bioavailability is the use of selective inhibitors of carnosinases (especially CNDP1), as this has been achieved using carnostatine in combination with carnosine [134]. Similarly, Anderson and collaborators described the rational design, characterization, and pharmacological evaluation of carnosinol, a derivative of carnosine with high oral bioavailability that is resistant to carnosinases [135]. By employing a rodent model of diet-induced obesity and metabolic syndrome, the authors demonstrated the ability of carnosinol to dose-dependently attenuate 4-hydroxynonenal adduct formation in liver and skeletal muscle, while simultaneously mitigating inflammation, dyslipidemia, insulin resistance, and steatohepatitis. As described by Grasso et al. [136], alternative approaches to increase carnosine delivery and its bioavailability include the use of carnosine derivatives [137,138,139]; vesicular systems (nanoliposomes, niosomes, and polymerosomes) [140,141,142,143]; and nanoparticulate systems [144,145,146,147]. The currently “under-used” intranasal administration route might also represent an innovative approach, since it can purportedly bypass the BBB and first-pass metabolism [148,149]; the vasodilatory activity of carnosine [150] also makes this endogenous dipeptide an attractive candidate for this kind of delivery. The intranasal administration of carnosine has been successfully adopted in two recent studies carried out by Bermúdez et al. in a Thy1-aSyn mouse model of PD characterized by the overexpression of human alpha-synuclein [148], the aberrant aggregation of which has been recognized as a key contributor to the neurodegenerative process observed in PD [151,152].

As previously mentioned, numerous in vivo studies have been carried out to demonstrate how exogenously administered carnosine can improve pathological conditions. However, the physiological role of carnosine and related histidine-containing dipeptides is not fully understood and should receive more attention. In this regard, substantial improvements could arise from the use of transgenic and knockout (KO) animal models. Very recently, Gonçalves et al. used a novel CARNS1 KO rat model to demonstrate that histidine dipeptides, including carnosine, are key regulators of excitation–contraction coupling in cardiac muscle [129]. Eckhardt and co-workers produced a mouse line deficient in CARNS1 and showed that CARNS1 deficiency is compatible with normal skeletal muscle and olfactory function but causes reduced olfactory sensitivity [153].

During the drafting of this position paper, a final point that drew my attention and that I would like to focus on relates to the use of anserine in combination with carnosine. What really surprised me is that, despite the fact that a large number of clinical trials have considered (or are still considering) this combination of molecules [79,82,83,84,92,94,154], only a few studies (I would say “close to zero”; see Qi et al. for an example almost impossible to find [155]) have investigated this kind of treatment at the preclinical level. Now, the question is “should clinical trials not be guided by preclinical ones?”. The obvious answer to this question should encourage us to think deeply about how we can more effectively connect and align future studies.

## 5. Concluding Remarks

Since the 1950s, more than a thousand research studies have been published on the structure, role, function, and biological activities of carnosine under numerous experimental and clinical conditions. As discussed above, a plethora of in vitro, in vivo, and clinical studies have been carried out showing the multimodal mechanism of action of carnosine, including anti-aggregant, anti-oxidant, and anti-inflammatory activities, which are all of great interest for numerous systemic and neurodegenerative disorders, such T2DM, PD, and AD. Despite this, as I attempted to underline in this position paper, advances need to be made in order to fully unveil the enormous therapeutic potential of this dipeptide, specifically in the context of in vivo studies, which are currently characterized by substantial heterogeneity regarding administration route, dosage, treatment duration, and animal model. The most urgent need is to perform pharmacokinetic studies to determine how the bioavailability of carnosine is connected to specific administration routes, dosages, and treatment durations. These studies will help to evaluate more accurately the existing results and plan future studies. Finally, there is also a need for “coordinated/aligned” preclinical studies laying the foundations for well-defined future clinical trials.

## Data Availability

Not applicable.
